# Characterization of the Composition of Bioactive Fractions from *Dendrobium officinale* Flowers That Protect against H_2_O_2_-Induced Oxidative Damage through the PI3K/AKT/Nrf2 Pathway

**DOI:** 10.3390/foods13193116

**Published:** 2024-09-29

**Authors:** Pengyan Zhu, Xinting Wang, XinLan Liu, Xiaojing Shen, Ai Li, Xiaohong Zheng, Jun Sheng, Wenjuan Yuan

**Affiliations:** 1 College of Science, Yunnan Agricultural University, Kunming 650201, China; zhupengyan16@163.com (P.Z.); 2013017@ynau.edu.cn (X.S.); 15087199742@163.com (A.L.); 15758205699@163.com (X.Z.); 2 Key Laboratory of Puer Tea Science, Ministry of Education, Yunnan Agricultural University, Kunming 650201, China; 18787628093@163.com (X.W.); liuxinlan15@163.com (X.L.); shengj@ynau.edu.cn (J.S.); 3 College of Food Science and Technology, Yunnan Agricultural University, Kunming 650201, China

**Keywords:** DOF, composition, antioxidant activity, inflammation activity, network pharmacology, PI3K/Akt/Nrf2 signaling pathways

## Abstract

*Dendrobium officinale* flowers (DOF) have previously been established as a promising source of natural antioxidants, and it is ideally suited for processing to prepare functional foods and food additives. The precise extraction processes employed, however, can alter the composition and antioxidant properties of the resultant products, and the characteristic compounds associated with the active fractions prepared from DOF or their mechanisms of action have yet to be reported. To clarify the molecular mechanisms through which these active fractions function for the first time, chromatography was used to separate DOF extracts, yielding five fractions (Fr. (a—e)). Analyses of the antioxidant activity for these different fractions revealed that Fr. (d) presented with the most robust bioactivity. Levels of total flavonoids were then measured, revealing that antioxidant activity levels were positively correlated with total flavonoid content. Fr. (d) was found to contain 20 flavonoids in HPLC-Triple-TOF-MS/MS analyses. At the cellular level, Fr. (d) was found to induce increases in the levels of protective antioxidant factors (SOD and GSH-Px) while reducing the levels of reactive oxygen species (ROS), damage-associated factors (MDA, NO, TNF-α, IL-1β, and IL-6), and inducible nitric oxide synthase (iNOS) expression in C2C12 cells that had been stimulated with H_2_O_2_. These data thus provided support for Fr. (d) prevention of oxidative stress and inflammation. Network pharmacology analyses further suggested that Fr. (d) can help protect against oxidative stress through its effects on PI3K/Akt-related signaling activity. Fr. (d) was subsequently found to upregulate PI3K/Akt pathway-related proteins, nuclear transcription factor 2 (Nrf2), and heme oxygenase 1 (HO-1) in addition to suppressing Kelch-like epoxide-related protein 1 (Keap1) expression. In summary, Fr. (d) was found to suppress PI3K/Akt/Nrf2 pathway activation, ultimately alleviating inflammation and oxidative stress as predicted with a network pharmacology approach. Future studies aimed at clarifying the composition and mechanistic activity of DOF Fr. (d) will likely help establish it as a functional food capable of promoting health and longevity.

## 1. Introduction

Oxidative stress stemming from reactive oxygen species (ROS) accumulation in cells can cause harm to macromolecules, including DNA, lipids, and proteins. Such oxidative injury drives a variety of clinically relevant diseases, including inflammatory conditions [1]. Under normal physiological conditions, nonenzymatic antioxidants derived from dietary sources and endogenous antioxidant enzymes like glutathione peroxidase (GSH-Px), catalase (CAT), and superoxide dismutase (SOD) can neutralize ROS and maintain redox homeostasis. The nuclear factor (erythroid-2-derived)-related factor 2 (Nrf2) signaling axis controls the transcription of these enzymatic antioxidants [2]. The Nrf2 transcription factor is normally sequestered by Keap1 in the cytosol, but exposure to elevated levels of oxidative stress can result in the dissociation of the two proteins such that Nrf2 can translocate to the nucleus and promote the upregulation of various cytoprotective factors, including heme oxygenase 1 (HO-1) [3,4,5]. Nuclear Nrf2 translocation is also influenced by various protein kinases, such as PI3K, AKT, and ERK [6]. Excessive ROS production can also trigger an inflammatory response, with immune cell-derived nitric oxide (NO) production being linked to iNOS upregulation and the excessive production of inflammatory interleukin-6 (IL-6), IL-1β, and tumor necrosis factor-α (TNF-α), thereby causing damage to normal tissues [7].

Natural products can be a valuable resource for agents that can improve health at the cellular and molecular levels. *Dendrobium officinale* exhibits antioxidant activity owing to the many bioactive components found in different parts of these plants [8,9]. As a traditional food and medicinal plant, *Dendrobium officinale*, has long been employed in traditional Chinese medicinal practices as the leader of “xiancao” [10,11]. *D. officinale* has a diverse array of detoxification, antidiabetic, antifibrotic, antitumor, antioxidant, anti-aging, immunomodulatory, and skin-protective properties [12,13,14,15,16,17,18]. Large-scale *D. officinale* cultivation production has fueled a dramatic rise in the availability of *D. officinale* flowers (DOF). DOF has traditionally been used to prepare teas, foods, and medicinal liquids, but the incomplete utilization of these preparations has led to undue research wastage. DOF is rich in nutrients, including amino acids, polyphenols, and flavonoids [19]. Given the abundant natural pigments found therein, DOF was approved as a new food ingredient by Chinese authorities in 2018.

The primarily bioactive antioxidants found in DOF include flavonoids and polyphenols such as quercetin, rutin, and merocyanine dye [20,21]. However, detailed studies on the chemical composition of DOF and the functions of different ingredients therein are lacking. In this study, an effort was made to optimize the solvent-based extraction of bioactive compounds from DOF and to quantify their antioxidant properties. Crude DOF extracts were prepared and separated via column chromatography (CC), yielding a series of fractions for which assessments of total flavonoid content and antioxidant activity were conducted. Of these, fraction d (Fr. (d)) exhibited higher levels of antioxidant activity. When Fr. (d) was characterized by HPLC-Triple-TOF-MS/MS, the metabolites therein were identified through comparisons with MS/MS spectra in the literature, offering insight into the chemical composition of Fr (d). The antioxidant capacity of Fr. (d) was also assessed through a network pharmacology approach and validated in H_2_O_2_-stimulated C2C12 cells.

## 2. Materials and Methods

### 2.1. Materials and Reagents

Dried *Dendrobium officinale* flowers were obtained from Pu’er City (Yunnan, China, batch no: 20210314). HaCat, SHSY5Y, and C2C12 cells were from the Kunming Wildlife Cell Bank of the Chinese Academy of Sciences (Kunming, Yunnan, China). Assay kits for Malondialdehyde (MDA, A003-1), glutathione (GSH, A006-2), and superoxide dismutase (SOD, A001-1) were from Nanjing Jiancheng Bioengineering Institute (Nanjing, China). 2,2′-azino-bis (3-ethylbenzothiazoline-6-sulfonic acid) (ABTS), 2,2-diphenyl-1-picrylhydrazyl (DPPH), and dexamethasone (DXMS) were from Solarbio Bioscience & Technology Co., Ltd. (Shanghai, China). Ethanol was from Tianjin Windship Chemical Reagent Technology Co., Ltd. (Tianjin, China). All other reagents were from Tianjin Damao Chemical Reagent Factory and were of analytical grade. High-glucose DMEM and fetal bovine serum (FBS) were from Gibco (New York, NY, USA), while 0.25% trypsin-EDTA, penicillin-streptomycin, MTT, RIPA lysis buffer, and PBS were from Solarbio (Beijing, China). A BCA assay kit was obtained from Biotopped (Beijing, China), while Nanjing Kaiji Biotechnology Development Co., Ltd. (Nanjing, China) was the source of a nuclear protein and cytoplasmic protein extraction kit (KGP150/KGP1100). Antibodies specific for AKT (A11016), p-AKT (AP1259), PI3K (A19742), p-PI3K (AP0854), Nrf2 (A0674), Keap1 (A1820), HO-1 (A1346), and Inos (A3774) were from ABclonal (Wuhan, China). Anti-β-tubulin (ab8227) was from Abcam (Waltham, MA, USA). Secondary antibodies (110191) were from R&D Systems (Minneapolis, MN, USA). Bio-Rad was the source of all Western blotting reagents (Davis, CA, USA). Any other reagents were of analytical grade and were purchased from local suppliers.

### 2.2. Crude Extracts Preparation

*Dendrobium officinale* flowers (DOF) were rinsed in their natural environment and air-dried for 3 days at 26 °C until reaching a final moisture content of <5%, after which they were pulverized using a laboratory crusher. These pulverized DOF samples were then passed through an 80-mesh screen, and the resultant powder (5.31 kg) was extracted using 70 L of 50% ethanol by heating samples for 4 h at 50 °C. The extract was then obtained by filtering this solution and concentrating it using a rotary evaporator, yielding 151 g of crude extract. This crude extract, in turn, was passed through a D101 macroporous resin column and eluted using a 1:1 water and ethanol (1500 mL each) gradient, producing five thin-layer chromatography (TLC) fractions, including the following: Fr. (a) 0.27 g (1:0), (b) 2.26 g (1:0–8:1), (c) 4.72 g (8:1–4:1), (d) 5.83 g (4:1–2:1), (e) 1.53 g (2:1–0:1).

### 2.3. Determination of Total Flavonoid Contents

Total flavonoid levels were measured using a slightly modified version of an approach that has been described previously [22]. Briefly, 1 mL from each sample extract was combined with 4 mL of deionized water and 300 μL 5% NaNO_2_ and equilibrated for 5 min, and 300 μL of 10% Al (NO)_3_ in methanol was next added. After resting for 6 min, 2 mL of 4% NaOH was added, and the volume was increased to 10 mL using water. Absorbance at 510 nm was quantified with a UV-Vis spectrophotometer. Flavonoid content in DOF samples was measured with a catechol standard curve, and the resultant measurements were reported in mg catechol equivalents/100 g extract dry weight (mg CE/100 g DW).

$$Y(\%)=\frac{C\times V\times N}{M}$$

where *Y* is the flavonoid extraction rate, *C* is the flavonoid content (mg/mL), *V* is the final solution volume (mL), *N* is the dilution factor, and *M* is the sampling volume for the flavonoid sample (mg).

### 2.4. UPLC-Q-Orbitrap HRMS Analysis

A sample stock solution was prepared through the dissolution of each standard at 1 mg/mL in methanol and incubating it for 15 min at 4 °C. Supernatants were filtered with a 0.22 μm nylon microporous filtration membrane, followed by injection for analysis. Fr. (d) from the crude extracts was analyzed with an Agilent 1290 UHPLC (Agilent Technologies, Santa Clara, CA, USA) instrument connected to a reversed-phase column (50 × 2.1 mm i.d., 1.7 μm, Agilent, Santa Clara, CA, USA). The mobile phase was ammonium acetate and 0.5% formic acid in water (A) and methanol (B) with the following elution gradient (flow rate: 0.4 mL/min): 0–0.5 min, 5% B; 0.5–10 min, 5%–100% B; 10–12 min, 100% B; 12–12.1 min, 100%-5% B; 12.1–16 min, 5% B. An injection volume of 2 μL was used.

Flavonoids were detected in negative and positive ion mode with a Nexera Triple-TOF/MS system (Nexera, Tokyo, Japan) equipped with an ESI ion source (AB Sciex, Redwood, CA, USA). A Triple-TOF/MS system was used for the collection of primary and secondary mass spectral data under the control of the software with the IDA function (Analyst TF 1.7, AB Sciex). The following source parameters were utilized: nebulizer gas (GS1; GS2): 60 psi; collision Energy: 35 ± 15 eV; electrospray capillary voltage: 5500 V; source temperature: 600 °C. Mass spectra were collected in the 60–1000 m/z range in negative and positive ion modes.

### 2.5. In Vitro Antioxidant Activity Analysis

#### 2.5.1. DPPH Assay

DPPH activity was quantified with a slightly modified version of methods reported previously [23]. Briefly, 0.3 mM DPPH in anhydrous ethanol was mixed with samples at a 1:1 (*v*/*v*) ratio, mixed thoroughly, and incubated without light for 30 min at 37 °C, after which absorbance at 517 nm was analyzed.

#### 2.5.2. Hydroxyl Radical Assay

Hydroxyl radicals were analyzed using a slightly modified version of methods that had been reported previously [24]. Briefly, equal amounts of sample solution, ferrous sulfate solution (6.0 mM), salicylic acid solution (10.0 mM), and H_2_O_2_ (10.0 mM) were combined, mixed thoroughly, and incubated in the dark for 30 min at 37 °C, after which absorbance at 510 nm was analyzed.

#### 2.5.3. ABTS Assay

An ABTS assay was conducted with a slightly modified version of the methods reported previously [25]. Briefly, the ABTS working solution was combined with samples at a 1:1 (*v*/*v*) ratio, followed by incubation without light for 30 min at 37 °C, followed by the measurement of absorbance at 734 nm.

#### 2.5.4. FRAP Assay

A FRAP assay was performed with a slightly modified version of the methods reported previously [26]. The FRAP reagent was compared by combining 0.3 M acetate buffer (pH 3.6), 10 mM TPTZ (tripyridyltriazine), and ferric chloride at a 10:1:1 (*v*/*v*/*v*) ratio, after which 200 µL of this reagent was mixed with 20 µL of prepared samples for 10 min at 25 °C, followed by the measurement of absorbance at 593 nm.

### 2.6. Network Pharmacology

DOF-related target information was obtained from the PubChem database, and the STRING database was used to develop a protein–protein interaction (PPI) network. The DAVID database was used for target gene enrichment analyses, and target pathways were visualized with Cytoscape 3.8.2.

### 2.7. Analyses of Fr. (d) Antioxidant and Anti-Inflammatory Activity

#### 2.7.1. Cell Culture

DMEM with 10% FBS and 1% PS was employed to culture C2C12 cells in a 37 °C humidified 5% CO_2_ incubator. When cells were confluent, they were subcultured with a trypsin solution. Routine cell passaging was performed every 1–2 days, and cells were used for subsequent experiments in the logarithmic growth phase.

#### 2.7.2. MTT Assay

An MTT (China Beijing Solaibao Technology Co., Ltd., Beijing, China) assay was used to assess viability. Briefly, absorbance at 492 nm was measured after a 24 h incubation with a microplate reader, as directed by the kit instructions.

#### 2.7.3. H_2_O_2_-Induced Modeling of Cell Injury

After allowing C2C12 cells to adhere to 96-well plates (5 × 10^5^ cells/mL), medium was exchanged for medium containing a range of H_2_O_2_ concentrations for 24 h, with the viability in each group then being analyzed to determine the best H_2_O_2_ dose for the modeling of oxidative injury.

#### 2.7.4. Intracellular ROS Detection

A Reactive Oxygen Species Assay Kit (Biyuntian Biotechnology Co., Ltd., Shanghai, China) was employed to analyze ROS levels using an approach that had been reported previously. Briefly, C2C12 cells were added to 6-well plates (5 × 10^5^ cells/mL), followed by drug treatment as above. At appropriate time points, a 20 min incubation with 1 mL of DCFH-DA (10 µM) was performed at 37 °C, followed by three washes with serum-free culture medium. A fluorescence microscope (Leica, Wetzlar, Germany) was then used to detect ROS levels.

#### 2.7.5. Analyses of MDA, SOD, and GSH-Px Activity

Following pretreatment using the same conditions as in the ROS analysis experiments, cells were harvested, ultrasonicated five times in an ice water bath (300 W, 5 s), and supernatants were then obtained through centrifugation (640× *g*, 10 min, 4 °C), after which they were used to analyze SOD, GSH-Px, and MDA content.

#### 2.7.6. NO and Cytokine Analyses

After appropriate cell modeling and treatment, the cells were lysed for 30 min with RIPA buffer and protease inhibitors on ice. Following centrifugation, supernatants were tested to measure NO, TNFα, IL-6, and IL-1β levels with appropriate ELISA kits (ABclonal, Technology, Beijing, China).

#### 2.7.7. Western Immunoblotting

After treating cells as appropriate, they were lysed in RIPA buffer, and a BCA assay kit was employed to measure protein levels in lysates. Samples were separated by SDS-PAGE and transferred onto PVDF membranes. These blots were blocked for 1 h with 5% skim milk, followed by overnight incubation with primary antibodies for the detection of AKT (1:500), p-AKT (1:500), PI3K (1:500) p-PI3K (1:1000), Nrf2 (1:1000), HO-1 (1:1000), iNOS (1:750), Keap-1 (1:1000), and β-tubulin (1:3000) at 4 °C. After a 1 h incubation with secondary antibodies (1:1500), blots were rinsed thrice with TBST (5 min/wash), and bands were detected with the ECL reagent and a detection system. Densitometric analyses were performed in ImageJ (NIH, Stapleton, NY, USA).

### 2.8. Statistical Analyses

Results are reported as means ± SD, with comparisons being made with unpaired one-tailed Student’s *t*-tests in GraphPad Prism (v 8.0.2), which was used for figure construction. *p* < 0.05 was regarded as significant. Western immunoblotting results are means ± SD from three independent measurements taken using ImageJ.

## 3. Results

### 3.1. Characterization of the Composition and Bioactivity of DOF Crude Extract and Fraction Samples

#### 3.1.1. Total Flavonoid Levels in Crude Extract and Fraction Samples

**Figure 1:**
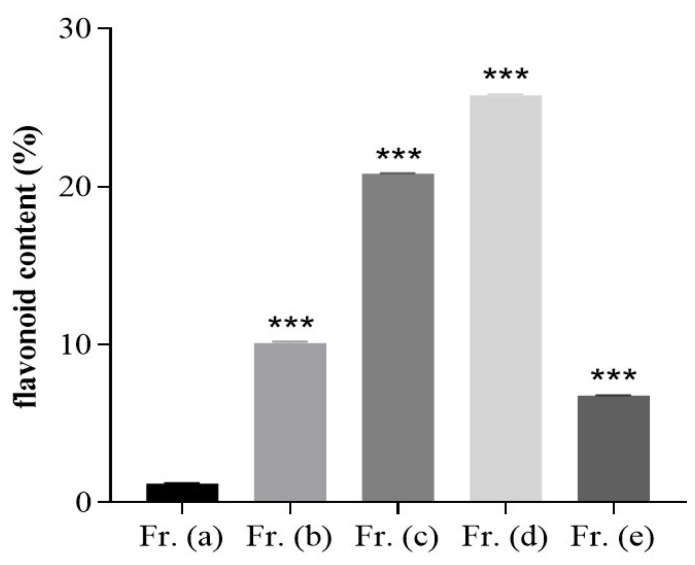
Evaluation of the total flavonoid content in five fractions (Fr. (a–e)). Data are means ± SD (*n* = 3). *** *p* < 0.001 vs. Fr. (a).

Flavonoids have been established as the main bioactive compounds present in DOF. In an effort to improve the isolation of these flavonoids, DOF extracts were prepared using various ethanol concentrations, ultimately yielding five fractions as identified through a TLC analysis designated Fr. (a–e). The total flavonoid content in the crude DOF fraction was analyzed using a standard curve prepared using rutin (R^2^ = 0.9991) (Figure S1). These five fractions exhibited varying flavonoid content levels, among which Fr. (d) exhibited the highest total flavonoid content at 25.77 ± 0.06% (Figure 1).

#### 3.1.2. Evaluation of Fraction-Specific Antioxidant Activity

Flavonoids extracted from DOF have previously been shown to exhibit significant antioxidant activity [27]. Accordingly, Fr. (b), (c), and (d) were next tested in DPPH, ABTS, FRAP, and hydroxyl radical assays.

**Figure 2:**
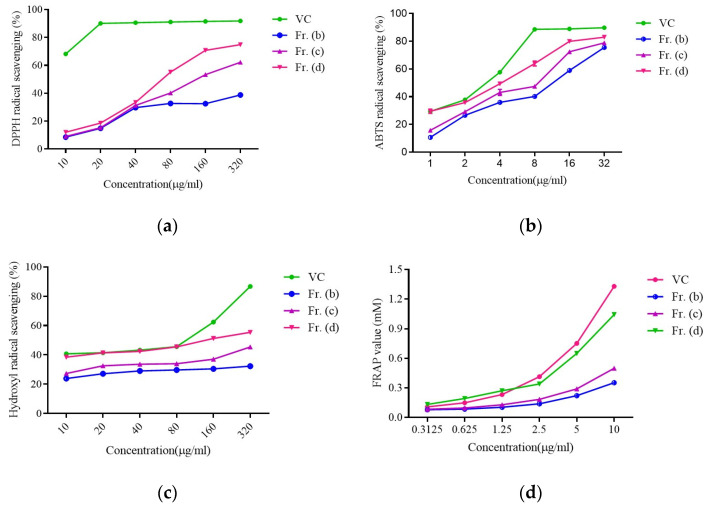
The impact of Fr. (**b**–**d**) and Vitamin C (Vc; positive control) on the scavenging of (**a**) DPPH, (**b**) ABTS, (**c**) Hydroxyl, and (**d**) FRAP radicals.

All three samples exhibited dose-dependent increases in DPPH scavenging activity, with vitamin C (Vc, positive control) exhibiting a maximum scavenging rate at a 20 µg/mL dose. As shown in Figure 2a, Fr. (d) presented with the strongest DPPH scavenging activity of the tested fractions (EC_50_ = 55 μg/mL). Similar results were also noted in the ABTS, FRAP, and hydroxyl radical scavenging assays (Figure 2b–d), and corresponding EC50 values are presented in Figure S2. Hydroxyl radical scavenging activity rose dose-dependently for all three fractions, with Fr. (d) exhibiting the highest rate (Figure 2b). Moreover, Fr. (d) exhibited the highest ABTS scavenging rate in the 2–32 μg/mL range (Figure 2b), while Fr. (d) exhibited the strongest FRAP activity in the 2.5–10 μg/mL range, with a FRAP value of 1.04 ± 0.01 mM at a Fr. (d) concentration of 10 µg/mL (Figure 2d), consistent with activity levels comparable to those for Vc. These findings offer clear support for the robust antioxidant activity of Fr. (d), such that it was selected for further study as a promising natural antioxidant.

#### 3.1.3. LC-MS Characterization of Flavonoids in DOF

**Figure 3:**
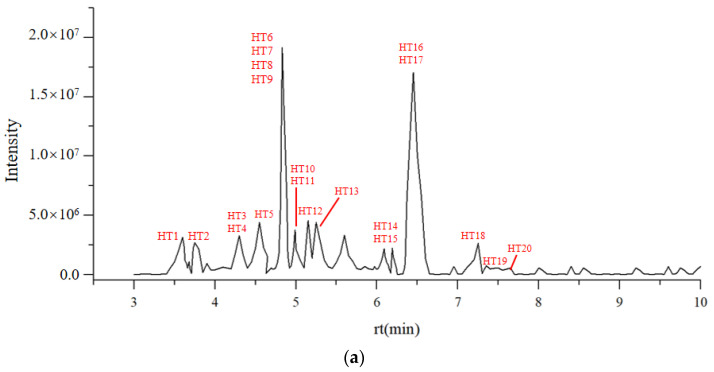

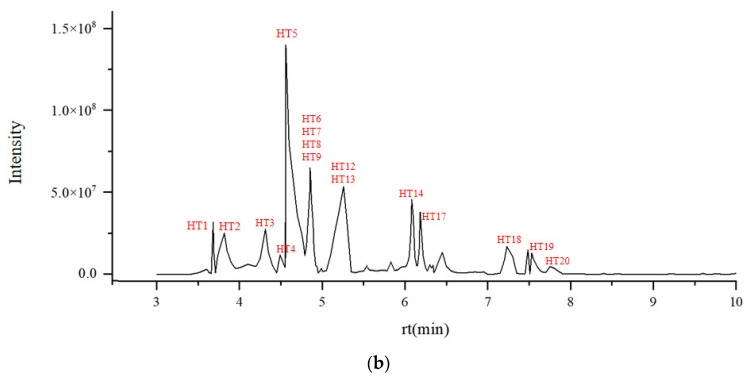
Chromatogram of (**a**) total ion current-positive and (**b**) total ion current-negative of 20 flavonoid compounds.

Given that the antioxidant activity of Fr. (d) was superior to that of the other analyzed fractions, and it exhibited a total flavonoid content of 25.77 ± 0.06%, a UHPLC-Triple TOF-MS/MS was next used to characterize the compounds present in this fractions in positive and negative ionization modes (Figure 3). Secondary product ions were used to obtain second-order fragment ions for corresponding mass values. Flavonoids present in Fr. (d) were identified based on the corresponding cracking law-matching method for the peaks containing MS2 ion fragments. Through comparisons of these results with prior publications and mass databases generated using MS data from over 2000 Chinese herbal medicine compounds, 20 total flavonoids were identified in these crude extracts (Table 1).

### 3.2. Network Pharmacology Analysis

**Figure 4:**
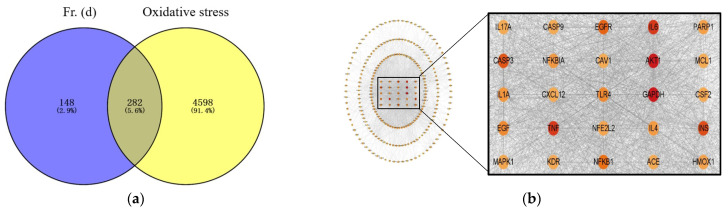

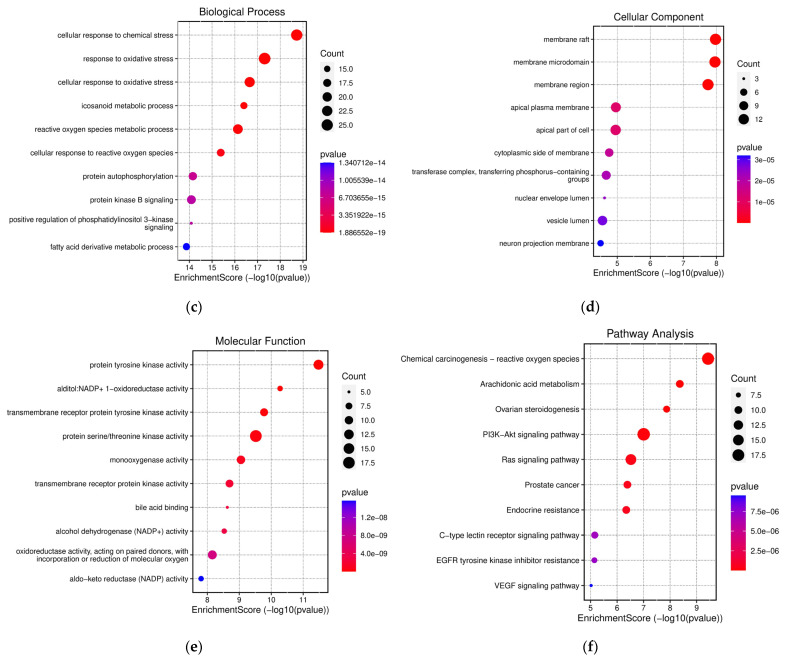

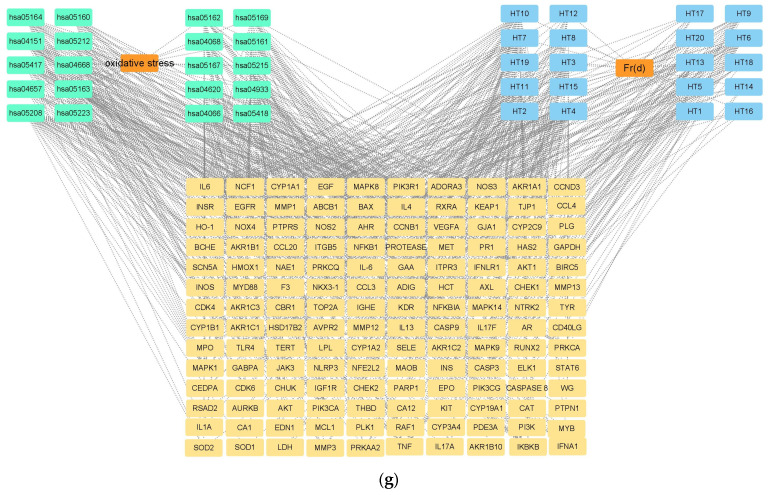
Network pharmacology analyses of the antioxidant activity of Fr. (d). Potential targets and active compounds associated with the antioxidant activity of Fr. (d) were explored with a network pharmacology analysis. (**a**). Intersecting targets linking Fr. (d) and oxidative stress; (**b**). The established PPI network for intersecting targets; (**c**–**e**) Biological process (C), cellular component (D), and molecular function (E) GO enrichment analysis; (**f**). KEGG pathway enrichment analysis; (**g**). “Ingredient-target-pathway-constructions. Disease pathways, active ingredients, and targets are shown in green, blue, and yellow, respectively.

In total, 20 flavonoids and 282 intersecting target proteins were identified using PubChem (Figure 4a). In the constructed network, line thickness is related to the strength of interactions between targets. Key targets associated with DOF antioxidant activity included AKT1, EGFR, IL6, caspase-3, NFKB1, HMOX1, EGF, INS, GAPDH, and TNF (Figure 4b). In GO biological process (BP), molecular function (MF), and cellular component (CC) enrichment analyses of hyperoside/quercetin target proteins (Figure 4c–e), results were presented by showing the proportion of all genes associated with a given term among the analyzed gene set, while bubble size is proportional to the number of genes and colors are proportional to *p*-values. GO-BP results suggested that Fr. (d) negatively regulates ROS metabolism and controls oxidative stress responses (Figure 4c). GO-CC results revealed that the compounds in Fr. (d) affect targets in the nucleus, membrane, and cytosol (Figure 4d). GO-MF findings suggested that Fr. (d) primarily functions through kinase binding (Figure 4e). In KEGG enrichment analyses, Fr. (d) was predicted to affect the PI3K-AKT pathway (Figure 4f). In Figure 4g, all Fr. (d) targets are presented on a purple background, while Nrf2 targets are presented on a blue background, and the related pathways are shown in red. The “Fr (d)-target-pathway” correlation analyses revealed that the antioxidant-related genes were primarily involved in the Nrf2, TNF, and AKT pathways. Under oxidative stress conditions, Nrf2 undergoes nuclear translocation to stimulate ARE-dependent gene transcription, leading to the upregulation of targets, including SOD, CAT, and HO-1. As such, Fr. (d) may exert its antioxidant effects primarily through its impact on PI3K/AKT/Nrf2 signaling.

### 3.3. Analyses of Toxicity

**Figure 5:**
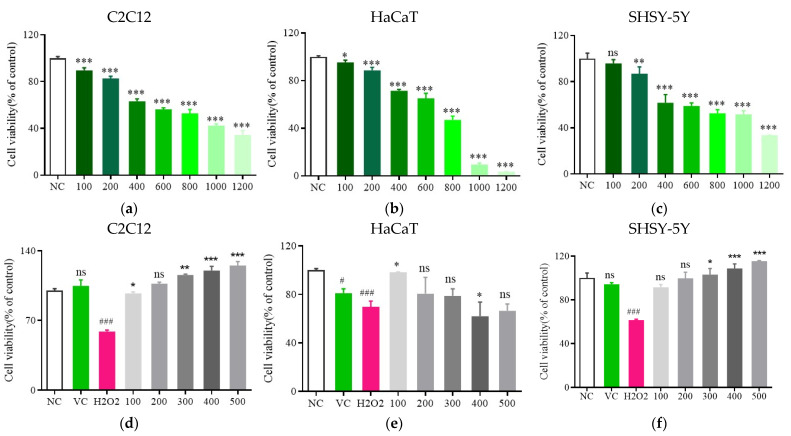
Analysis of the toxic effects of Fr. (d). (**a**–**c**). C2C12, HaCaT, and SHSY-5Y cell viability in response to H_2_O_2_ treatment; (**d**–**f**). Fr. (d) protects against H_2_O_2_-induced oxidative damage in the C2C12, HaCaT, and SHSY-5Y cells. Data are means ± SD (*n* = 6). # *p* < 0.05, and ### *p* < 0.01 vs. NC group; *** *p* < 0.001, ** *p* < 0.01, * *p* < 0.05, ^ns^ *p* > 0.05 vs. VC.

H_2_O_2_ exposure can cause significant cytotoxicity, inducing senescence and oxidative damage through its effects on cellular ROS production [42]. For this study, the impact of H_2_O_2_ on the viability and proliferation of HaCaT, C2C12, and SHSY-5Y cells was assessed, revealing a dose-dependent decline in cellular viability with increasing H_2_O_2_ concentrations. Following treatment with 600 μmol/L H_2_O_2_, HaCaT, C2C12, and SHSY-5Y viability was significantly reduced to (65.2 ± 5.06)%, (56.36 ± 1.57)%, and (59.03 ± 3.22)%, respectively (*p* < 0.05, Figure 5a–c). This H_2_O_2_ concentration was thus selected for subsequent experiments testing the protective effects of Fr. (d) treatment.

Following Fr. (d) treatment at varying doses for 24 h, a significant decrease in oxidative injury to all three tested cell lines was observed if cells were treated with Fr. (d) at 100–500 μg/mL (*p* < 0.05, Figure 5d–f). Notably, Fr. (d) effectively prevented H_2_O_2_-induced cell death in this experimental system (*p* < 0.05). Flavonoid concentrations in the 100–500 μg/mL are thus capable of reversing H_2_O_2_-associated damage, with the most pronounced effects in the 200–400 μg/mL range. As the C2C12 cells presented with the greatest sensitivity in this system, they were selected as the model for subsequent mechanistic work, which was performed using three Fr. (d) doses (200, 300, and 400 μg/mL).

### 3.4. Fr. (d) Protects C2C12 Cells against H_2_O_2_-Induced Injury

**Figure 6:**
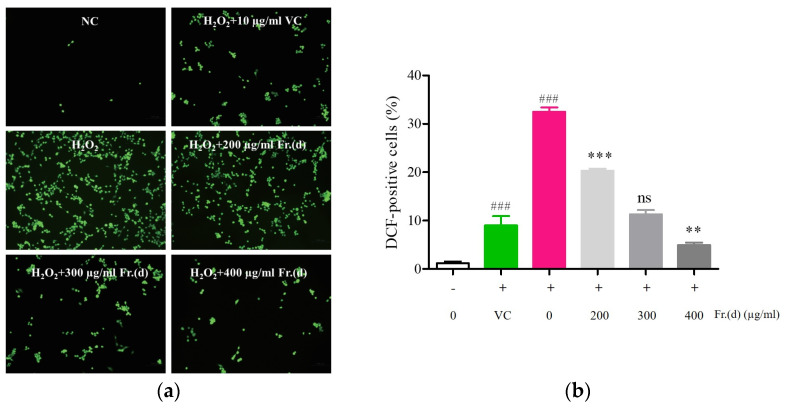
Fr. (d) protects against H_2_O_2_-induced oxidative stress. C2C12 cells were treated with H_2_O_2_ and Fr. (d) (200–400 µg/mL). (**a**). ROS in each group were detected via fluorescent imaging. (**b**). Fluorescence signal intensity. Data are means ± SD (*n* = 3). ### *p* < 0.01 vs. NC group; *** *p* < 0.001, ** *p* < 0.01, ^ns^ *p* > 0.05 vs. Vc.

DCF-DA fluorescence was next measured to indirectly assess ROS concentrations in different samples, revealing significantly greater fluorescence intensity in H_2_O_2_-treated cells (32.47 ± 0.92%) relative to controls (1.19 ± 0.38%) (*p* < 0.005, Figure 6a,b), supporting the induction of ROS accumulation in these cells. Fr. (d) dose-dependently reduced the fluorescence within these cells relative to those in H_2_O_2_-treated cells (*p* < 0.05). High concentrations of Fr. (d) exhibited reductions in ROS levels (4.94 ± 0.53%) relative to positive control Vc treatment (9.00 ± 1.95%, *p* < 0.05). Fr. (d) is thus capable of effectively inhibiting increases in ROS levels within C2C12 cells exposed to H_2_O_2_, abrogating associated oxidative damage.

**Figure 7:**
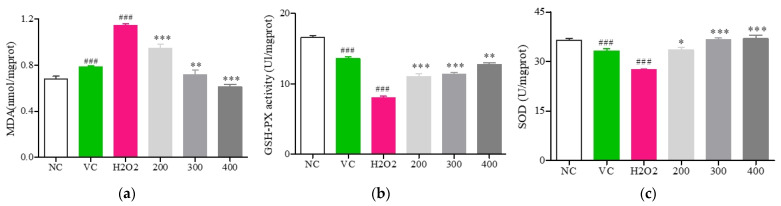
The impact of DOF-derived flavonoids on H_2_O_2_-induced oxidative stress. (**a**) MDA; (**b**) GSH-Px; (**c**) SOD. Data are means ± SD (*n* = 3). ### *p* < 0.01 vs. NC group; *** *p* < 0.001, ** *p* <0.01 and * *p* < 0.05 vs. H_2_O_2_ treatment.

To test the ability of Fr. (d) to mitigate H_2_O_2_-induced oxidative stress, a series of experiments investigating antioxidant enzymes and lipid peroxidation were next performed (Figure 7). Levels of MDA within H_2_O_2_-treated cells rose to 1.14 ± 0.02 nmol/mg (*p* < 0.01, Figure 7a), consistent with the induction of lipid peroxidation. However, the treatment of these cells with Fr. (d) at concentrations of 200, 300, and 400 μmol/L significantly reduced MDA levels to 0.94 ± 0.04, 0.72 ± 0.04, and 0.61 ± 0.02 nmol/mg, respectively. Positive control Vc treatment decreased MDA levels to 0.78 ± 0.01 nmol/mg. At intermediate and high concentrations, Fr. (d) outperformed Vc in terms of its ability to reduce MDA levels (*p* < 0.05).

Relative to control cells, those stimulated with H_2_O_2_ exhibited a significant reduction in GSH-Px activity to 8.05 ± 0.2 U/mg (*p* < 0.05; Figure 7b). However, GSH-Px activity levels following treatment with Fr. (d) at 200, 300, and 400 μmol/L were significantly increased to 11.04 ± 0.38 U/mg, 10.94 ± 0.31 U/mg, and 12.75 ± 0.27 U/mg (*p* < 0.05), respectively. GSH-Px activity in the Vc group (13.6 ± 0.26 U/mg) was higher than that in the Fr. (d) groups.

SOD activity was reduced to 27.45 ± 0.21 U/mg following H_2_O_2_ treatment (*p* < 0.05, Figure 7c), consistent with the ability of H_2_O_2_ to disrupt endogenous antioxidant activity and to induce oxidative stress. Significantly higher SOD activity levels were observed in the Fr. (d) (200, 300, and 400 μmol/L) groups relative to those in cells treated with H_2_O_2_ (*p* < 0.05), and SOD activity in cells treated using 300 μmol/L Fr. (d) was higher compared with Vc-treated cells (*p* < 0.05). Fr. (d) thus exhibits robust antioxidant activity, can protect against lipid peroxidation, and coordinates the induction of robust antioxidant defenses.

### 3.5. Examination of the Anti-Inflammatory Impact of Fr. (d) on H_2_O_2_-Exposed C2C12 Cells

**Figure 8:**
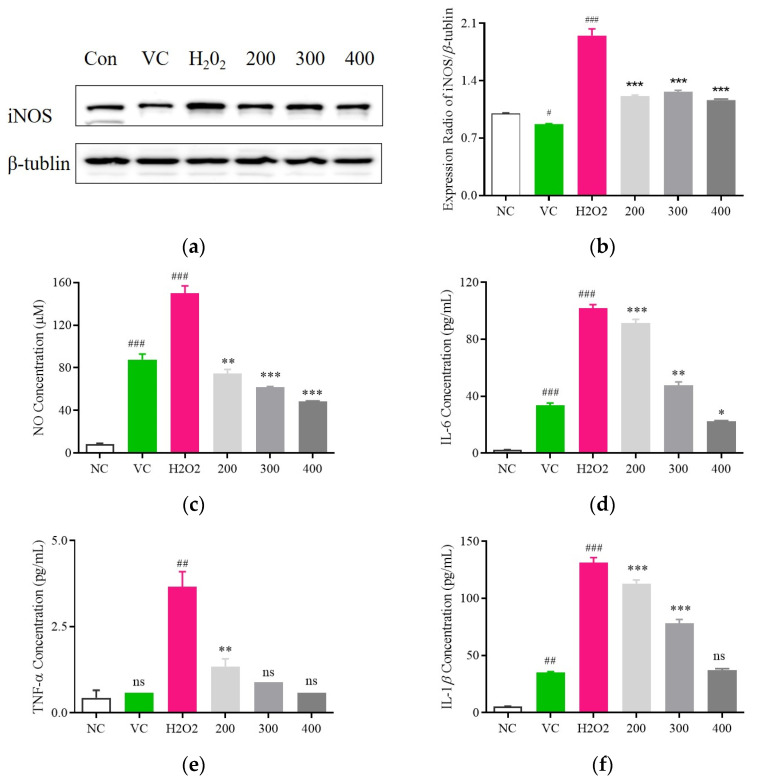
Effects of Fr. (d) on the release of inflammation factors in H_2_O_2_ model. After the intervention, the levels of iNOS (**a**,**b**), NO (**c**), IL-6 (**d**), TNF-α (**e**), and IL-1β (**f**) in the H_2_O_2_-induced C2C12 cell were detected to evaluate the effects of Fr. (d) on inflammation. The data shown represent the mean ± SD (*n* = 3). ## *p* < 0.01, ### *p* < 0.001 when compared with control versus H_2_O_2_; *** *p* < 0.001, ** *p* < 0.01, * *p* < 0.05, ^ns^ *p* > 0.05 as compared to the group treated with H_2_O_2_ alone.

To clarify the anti-inflammatory effects associated with Fr. (d) treatment, iNOS expression was analyzed in H_2_O_2_-treated C2C12 cells. Significantly reduced iNOS levels were evident in these H_2_O_2_-induced cells following Fr. (d) treatment, with Vc serving as a positive control (Figure 8a,b). Levels of the key inflammatory mediators NO, TNFα, IL-1β, and IL-6 were increased by 17.65-fold, 8.32-fold, 17.48-fold, and 13.1-fold, respectively, in the model group relative to controls (Figure 8c–f). However, treatment with Fr. (d) (200, 300, and 400 μmol/L) led to significant reductions in the levels of NO to 74.41 ± 1.08 μM, 61.78 ± 0.71 μM, and 48.39 ± 0.6 μM, respectively, in line with the effects observed following positive control Vc treatment (87.64 ± 5.25 μM) (Figure 8c). Similarly, these respective Fr. (d) doses reduced TNFα levels 1.35 ± 0.22 pg/mL, 0.89 ± 0.01 pg/mL, and 0.59 ± 0.01 pg/mL (Vc: 5.29 ± 0.49 pg/mL) (Figure 8d), reduced IL-1β levels to 113.06 ± 3.15 pg/mL, 78.14 ± 3.42 pg/mL and 37.02 ± 1.47 pg/mL (Vc: 87.64 ± 5.25 pg/mL) (Figure 8e), and decreased IL-6 levels to 91.42 ± 2.6 pg/mL, 47.66 ± 2.36 pg/mL and 22.25 ± 0.57 pg/mL (Vc: 33.6 ± 1.69 pg/mL) (Figure 8f). Fr. (d) may thus be capable of suppressing the production of inflammatory mediators at the transcriptional level.

### 3.6. Fr. (d) Suppresses Oxidative Stress by Activating the Akt/Nrf2 Axis

**Figure 9:**
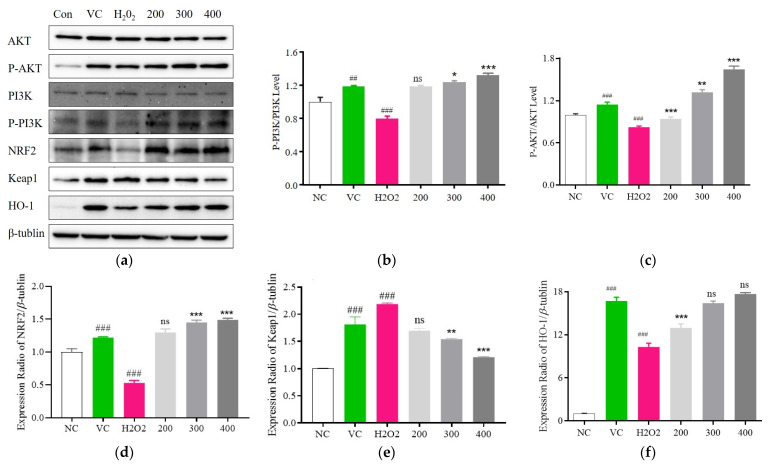
DOF-derived flavonoids impact Nrf2/HO-1-mediated antioxidant activity (**a**). Expression of related pathway proteins P-PI3K/PI3K (**b**), P-Akt/Akt (**c**), Nrf2 (**d**), Keap1 (**e**), HO-1 (**f**) was detected using Western blot. Data are means ± SD (*n* = 3). ### *p* < 0.001, ## *p* < 0.01when compared with control versus H_2_O_2_; *** *p* < 0.001, ** *p* < 0.01, * *p* < 0.05, ^ns^ *p* > 0.05 vs. H_2_O_2_ alone.

In network pharmacology analyses, Akt was identified as a key Fr. (d) target protein related to the inhibition of oxidative stress. To confirm this predicted relationship, PI3K-Akt signaling pathway protein levels were next analyzed (Figure 9a), revealing a pronounced reduction in PI3K and Akt in the H_2_O_2_-stimulated model group relative to control cells, while Fr. (d) (200, 300, and 400 μmol/L) significantly reversed these changes in the p-PI3K/PI3K and p-Akt/Akt ratios (Figure 9b,c). Akt can also regulate Nrf2 expression, with Nrf2 undergoing nuclear translocation following its separation from Keap1 such that it can promote HO-1 upregulation to coordinate antioxidant responses. Here, significantly reduced Nrf2 and HO-1 levels were noted in the H_2_O_2_-treated model group relative to control cells, while Fr. (d) (200, 300, and 400 μmol/L) restored these expression changes while also reducing Keap1 expression, consistent with the induction of an antioxidant response (Figure 9d–f). Fr. (d) thus appears to exert its protective effects, at least in part through the regulation of PI3K/AKT/Nrf2 signaling, ultimately protecting cells against oxidative injury.

## 4. Discussion

This study developed a green and efficient process for the extraction of total flavonoids, allowing full utilization of DOF flavonoids. Using the total flavonoid yield of DOF as an indicator, different concentrations of solvents were used for elution on a macroporous adsorption resin to obtain the extraction fractions. The D101 macroporous resin showed good adsorption [43] and was thus used to adsorb flavonoids from DOF, while ethanol was used for desorption of the saturated resin to determine flavonoid content. In the study, the flavonoids were extracted, followed by preliminary purification, with Fr. (d) exhibiting the highest total flavonoid content of 25.77 ± 0.06%. Compared with traditional organic solvents, this method is both more efficient and environmentally friendly, and the solvent can be reused, indicating its potential for use in industrial applications.

Flavonoids are known to be responsible for the antioxidant activity of DOF. Thus, to gain a better understanding of the relationship between the total flavonoid content and antioxidant activity in DOF extraction fractions, ABTS, DPPH, FRAP, and hydroxyl radical assays were used, showing that the total flavonoid content was positively correlated with the ABTS, FRAP, and DPPH scavenging activity, while hydroxyl radical assays indicated that higher total flavonoid contents were responsible for increased antioxidant activity. Nonetheless, due to the abundance of flavonoids in Fr. (d), the separation and identification of these compounds posed a significant challenge. Thus, the flavonoid compositions were examined by UPLC-QTOF-MS/MS. It was found that 50% of the flavonoids were flavonols. Many previous studies have shown that flavonols may be the key compounds contributing to the health-promoting functions of DOF [44].

Extensive research has shown an association between oxidative stress and a variety of diseases [1], making the prevention of oxidative stress a crucial property of functional foods. Cellular H_2_O_2_-induced oxidative injury models have become both well-established and commonly used in vitro methods for the evaluation of antioxidant activities [45]. H_2_O_2_, which is produced as a result of dopamine oxidation and enzymatic activity, can lead to oxidative stress and damage in cell lines by promoting the release of ROS. ROS production and elimination are in a state of dynamic homeostasis under physiological conditions. Exposure to deleterious stimuli, however, can disrupt this delicate balance, leading to excessive ROS accumulation and consequent damage to cellular membranes and macromolecules, including proteins, lipids, and DNA [46]. In cells exposed to H_2_O_2_, a positive association between Fr. (d) dose and cell viability was observed, consistent with the protective benefits of this fraction as a tool to mitigate H2O2-induced cell damage.

ROS homeostasis is highly dependent on the control of the antioxidant enzyme, with endogenous enzymes, including CAT, SOD, and GSH-Px, being essential to control ROS biogenesis through the conversion of these radicals into less damaging compounds [47]. Elevated levels of these enzymes may coincide with better protection against oxidative stress induced by H_2_O_2_ [48,49]. MDA levels in cells indicate the extent of cell damage and lipid peroxidation [50]. Under physiological conditions, cellular antioxidants, including SOD, CAT, and GSH, can defend against oxidative stress and inflammation to protect the organism. However, an imbalance between pro-oxidant and antioxidant factors exacerbates damage to both the cell and the organism.

The production of excessively high ROS levels can damage tissues. In addition to antioxidant enzymes, inflammatory mediators are central to the oxidative stress and inflammation that take place following H_2_O_2_ treatment. NO, TNFα, IL-1β, and IL-6 are the primary factors engaged in the context of H_2_O_2_-induced inflammation, and iNOS is central to the pathogenesis of inflammatory disease owing to its ability to enhance vascular permeability and contribute to the onset of tissue damage [51,52]. In this study, it was found that Fr. (d) could restore the balance between defensive and aggressive factors, inhibiting oxidative stress and inflammation and, thereby, attenuating H_2_O_2_-induced damage.

To further clarify the potential targets and pathways associated with the action Fr. (d), we constructed a drug-component-target network. The KEGG enrichment results showed that Fr. (d) could regulate redox homeostasis. PPI networks showed that antioxidant-related genes were primarily involved in the Nrf2, Akt, and TNF signaling pathways. Akt-mediated transcription activates the production of a series of enzymes promoting oxidative stress as well as proinflammatory factors to exacerbate cellular oxidative stress [6]. Nrf2 is a major transcription factor that plays a pivotal role in endogenous protection against oxidative stress. In response to oxidative stress, Nrf2 translocates to the nucleus, where it promotes the transcription of ARE-dependent genes, including NQO1, HO-1, CAT, and SOD [53]. In addition, Nrf2 is a downstream gene of the PI3K/Akt axis, which is known to regulate oxidative stress through Nrf2 and participate in the pathological processes of various diseases [54]. The Western blot results also confirmed the role of Fr. (d) in regulating the Nrf2/HO-1 pathway and downregulating Keap1 contents, as well as upregulating phosphorylation of key proteins in the PI3K-Akt signaling pathway.

This was the first study to evaluate the composition of Fr. (d) from DOF, together with its antioxidant and anti-inflammatory activities. The antioxidant activity of Fr. (d) was found to depend primarily on activation of the PI3K/Akt/Nrf2 signaling pathway. It is suggested that future studies should use metabolomics and proteomics in animal models for a better understanding of the actions and variations in different natural products and their antioxidant effects.

## 5. Conclusions

In summary, crude extracts of DOF were prepared and separated by column chromatography, ultimately yielding five fractions, among which Fr. (d) presented with the highest total flavonoid content and the most robust antioxidant activity. UHPLC-Triple TOF-MS/MS analyses revealed the presence of 20 flavonoids in Fr. (d), and these results offer the first evidence in favor of the cytoprotective effects of Fr. (d) through mechanisms that may be related to the control of oxidative stress and inflammation regulated by the PI3K/Akt/Nrf2 signaling axis. From a nutritional perspective, Fr. (d) may be an ideal candidate for the prevention of oxidative damage in cells. Together, these results thus offer a sound theoretical foundation for efforts to support the further utilization of DOF.

## Figures and Tables

**Table 1:** Major active compounds among total flavonoids in Fr. (d).

NO	ID	Compounds	RT (min)	Measured Value Exptl (*m*/*z*)	Theoretical Value Exptl (*m*/*z*)	Fragmentation (*m*/*z*)	References
1	HT 1	5,7-Dimethoxyflavanone	3.686	285.1218	285.1082	267.1123/195.0891	[28]
2	HT 2	5-Methoxyflavone	3.742	253.0943	253.0820	134.0943/207.0894/162.0914/117.0700/235.0896	[29]
3	HT 3	Rutin	4.300	610.151	610.1534	593.1523/503.1192/473.1105/399.0742/369.0615/301.0372/293.0483	[30]
4	HT 4	2′,3-Dihydroxy-4,4′,6′-Trimethoxychalcon	4.494	331.1113	331.1137	331.2293/331.2449/331.1526/331.1780/331.1316/285.1210/177.0579/178.0557/107.041	[31]
5	HT 5	Delphinidin 3-glucoside	4.565	465.093	465.1028	301.0369/300.0322	[32]
6	HT 6	Maysin	4.853	576.1438	576.1479	455.0999/341.0695	[32]
7	HT 7	Biorobin	4.853	594.1536	594.1585	285.0419/284.0330	[32]
8	HT 8	Cyanidin 3-O-glucoside	4.858	449.2615	449.1079	287.0548	[33]
9	HT 9	Puerarin	4.993	416.1056	416.1107	415.1983/463.1313/323.0758/295.0614/267.0638/161.0625/179.034 0/117.0759	[34]
10	HT 10	Astragalin	4.999	449.1617	449.1039	449.2500/287.0515	[32]
11	HT 11	Kaempferol-3-O-glucoside	5.257	448.1006	448.0960	284.0333/285.0406/255.0302/161.025	[35]
12	HT 12	Quercitrin	5.262	448.0955	448.1006	300.0291/301.0360/323.0776/271.0248/161.0253	[32]
13	HT 13	Isovitexin	5.567	432.0991	432.1056	431.1771/431.2180/431.0645/431.2556/431.1956/311.0560/251.1276/161.0249/89.0243	[36]
14	HT 14	Apigenin	6.091	270.0469	270.0528	269.1814/181.1223/72.9931	[37]
15	HT 15	Tricin	6.157	331.0805	331.0773	302.0384/331.2030/316.0507/315.453/331.1346/315.0570/270.0533/331.0191/165.0539/153.0077	[38]
16	HT 16	(2Z)-4,6-Dihydroxy-2-[(4-hydroxy-3,5-dimethoxyphenyl)methylidene]-1-benzofuran-3-one	6.184	330.0682	330.0740	313.0375/314.0444/299.0207/271.0251	[39]
17	HT 17	Chrysoeriol	6.506	301.0683	301.0667	286.0466/301.0298/258.0513/301.1205/301.0090/283.0991/86	[40]
18	HT 18	Chrysin	7.236	254.0517	254.0579	235.1676/209.0580	[32]
19	HT 19	Galangin	7.485	270.0456	270.0528	-	[32]
20	HT 20	Kaempferol	7.526	286.0444	286.0477	229.0522/107.0153	[41]

## Data Availability

The original contributions presented in the study are included in the article/supplementary material, further inquiries can be directed to the corresponding author.

## Supplementary Materials

The following supporting information can be downloaded at: https://www.mdpi.com/article/10.3390/foods13193116/s1; Figure S1: Rutin standard curve; Figure S2: Total ion chromatogram of Fr. (d); Figure S3: EC_50_ values for DPPH, ABTS, FRAP, and hydroxyl radical scavenging.
