# Supplementary material for: Characterization of the Composition of Bioactive Fractions from Dendrobium officinale Flowers That Protect against H2O2-Induced Oxidative Damage through the PI3K/AKT/Nrf2 Pathway
Source: Foods. 2024 Sep 29;13(19):3116. doi: 10.3390/foods13193116 (PMC11475059; doi:10.3390/foods13193116)

Supplementary documents

Characterization of the Composition of Bioactive Fractions from *Dendrobium officinale* Flowers That Protect against H_2O_2-Induced Oxidative Damage through the PI3K/AKT/Nrf2 Pathway

Pengyan Zhu [1,2,†], Xinting Wang [2,3,†], XinLan Liu [2,3], Xiaojing Shen [1], Ai Li [1], Xiaohong Zheng [1], Jun Sheng [2] and Wenjuan Yuan [1,2,*]

[1] College of Science, Yunnan Agricultural University, Kunming 650201, China; zhupengyan16@163.com (P.Z.); 2013017@ynau.edu.cn (X.S.); 15087199742@163.com (A.L.); 15758205699@163.com (X.Z.)
[2] Key Laboratory of Puer Tea Science, Ministry of Education, Yunnan Agricultural University, Kunming 650201, China; 18787628093@163.com (X.W.); liuxinlan15@163.com (X.L.); shengj@ynau.edu.cn (J.S.)
[3] College of Food Science and Technology, Yunnan Agricultural University, Kunming 650201, China
* Correspondence: yuanwj0805@126.com
† These authors contributed equally to this work.

Supporting information

Fig S1. Rutin standard curve.

Fig S2. Total ion chromatogram of Fr.(d).

Fig S2.1 Total ion chromatogram of Fr.(d) in POS modes.

Fig S2.2 Total ion chromatogram of Fr.(d) in NEG modes.

Fig S2.3 Total ion chromatogram of blank solvent in POS modes.

Fig S2.4 Total ion chromatogram of blank solvent in NEG modes.

Fig S3. EC50 values for DPPH, ABTS, FRAP and hydroxyl radical scavenging.

Fig S1. Rutin standard curve.

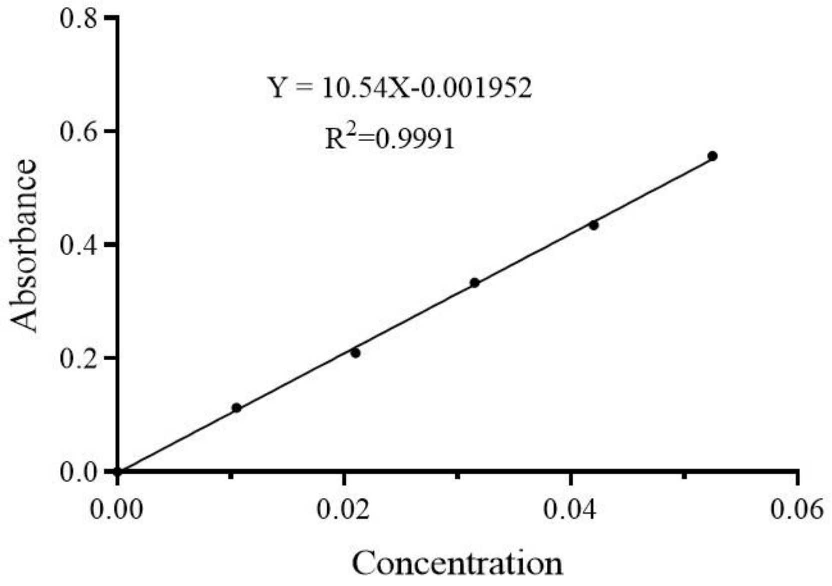

Fig S2. Total ion chromatogram of Fr.(d).

Fig S2.1 Total ion chromatogram of Fr.(d) in POS modes.

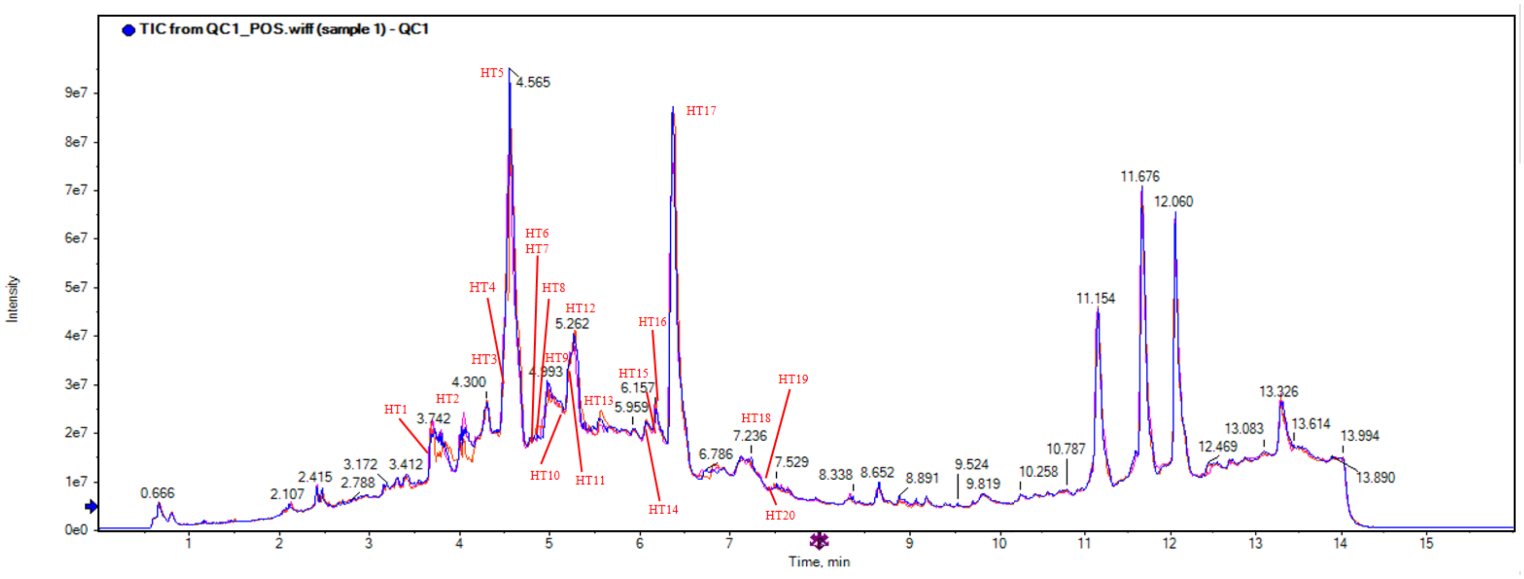

Fig S2.2 Total ion chromatogram of Fr.(d) in NEG modes.

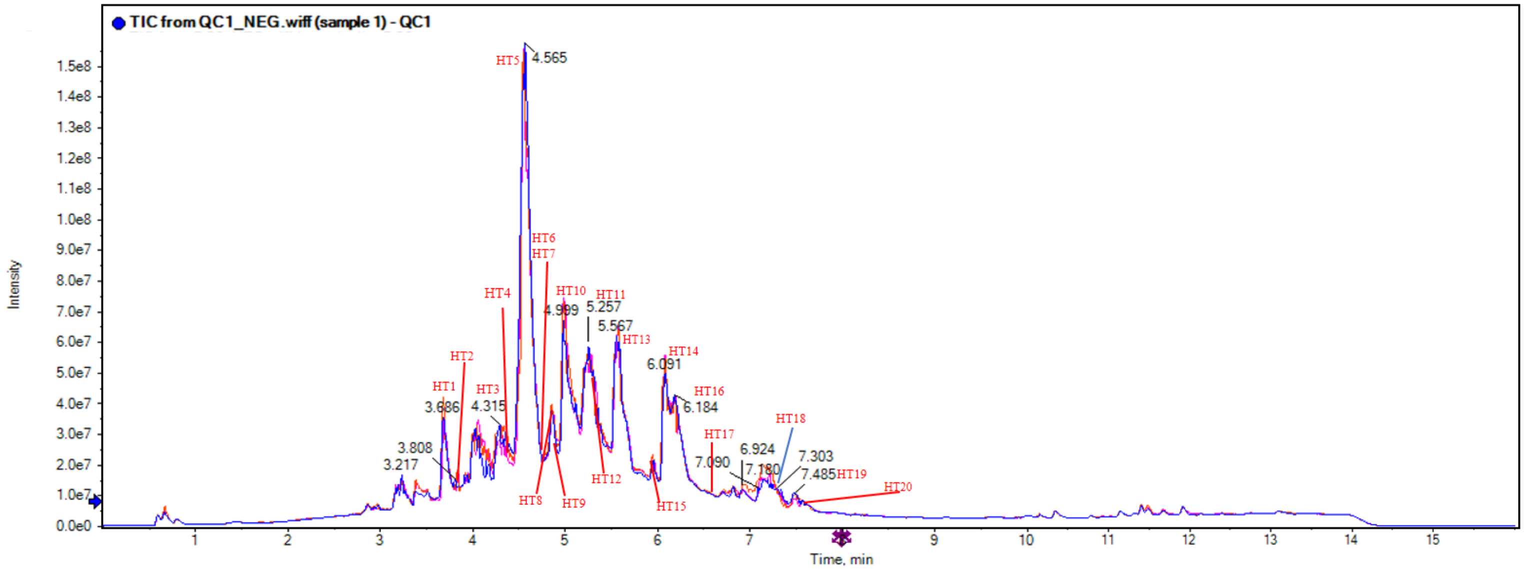

Fig S2.3 Total ion chromatogram of blank solvent in POS modes.

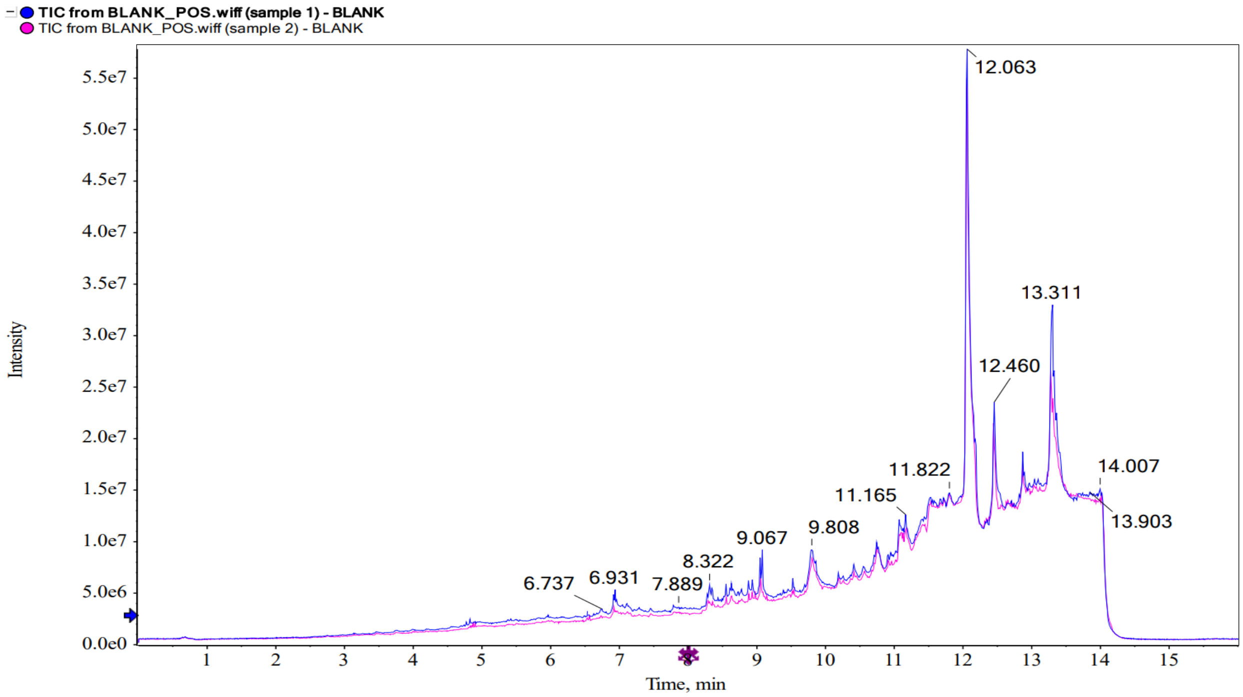

Fig S2.4 Total ion chromatogram of blank solvent in NEG modes.

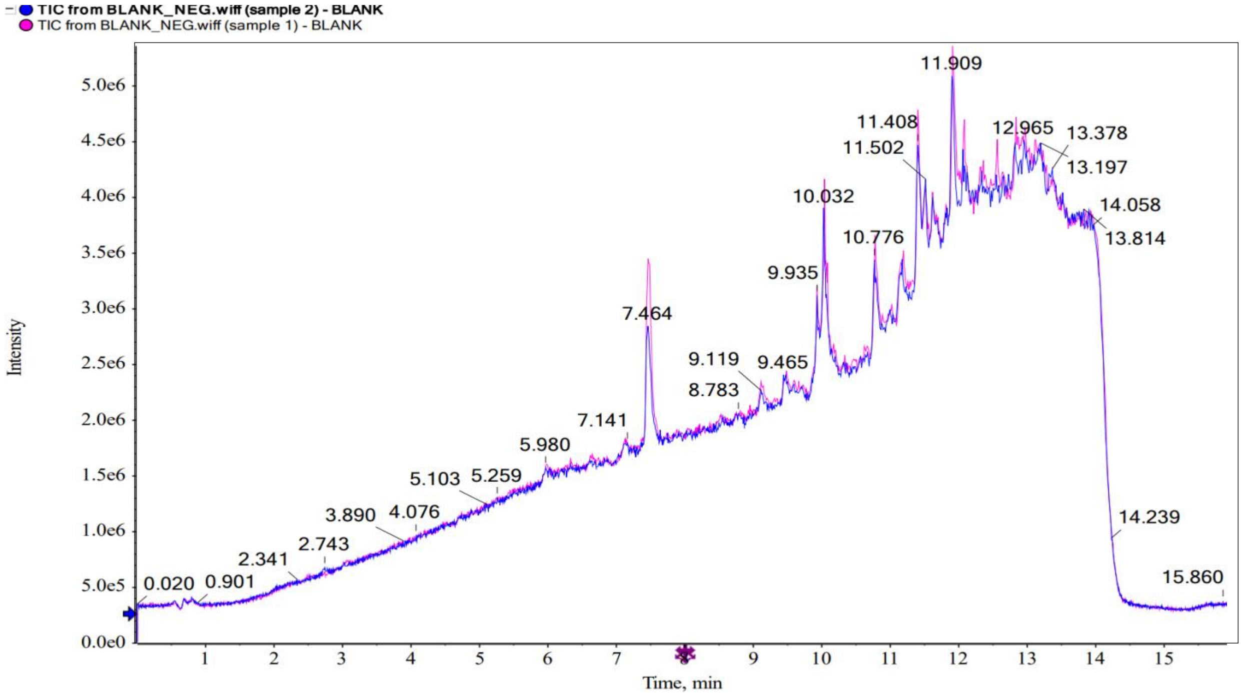

Fig S3 EC50 values for DPPH, ABTS, FRAP and hydroxyl radical scavenging, Data are means ± SD (n=3). *** p < 0.001, ** p <0.01, * p < 0.05 vs. VC.

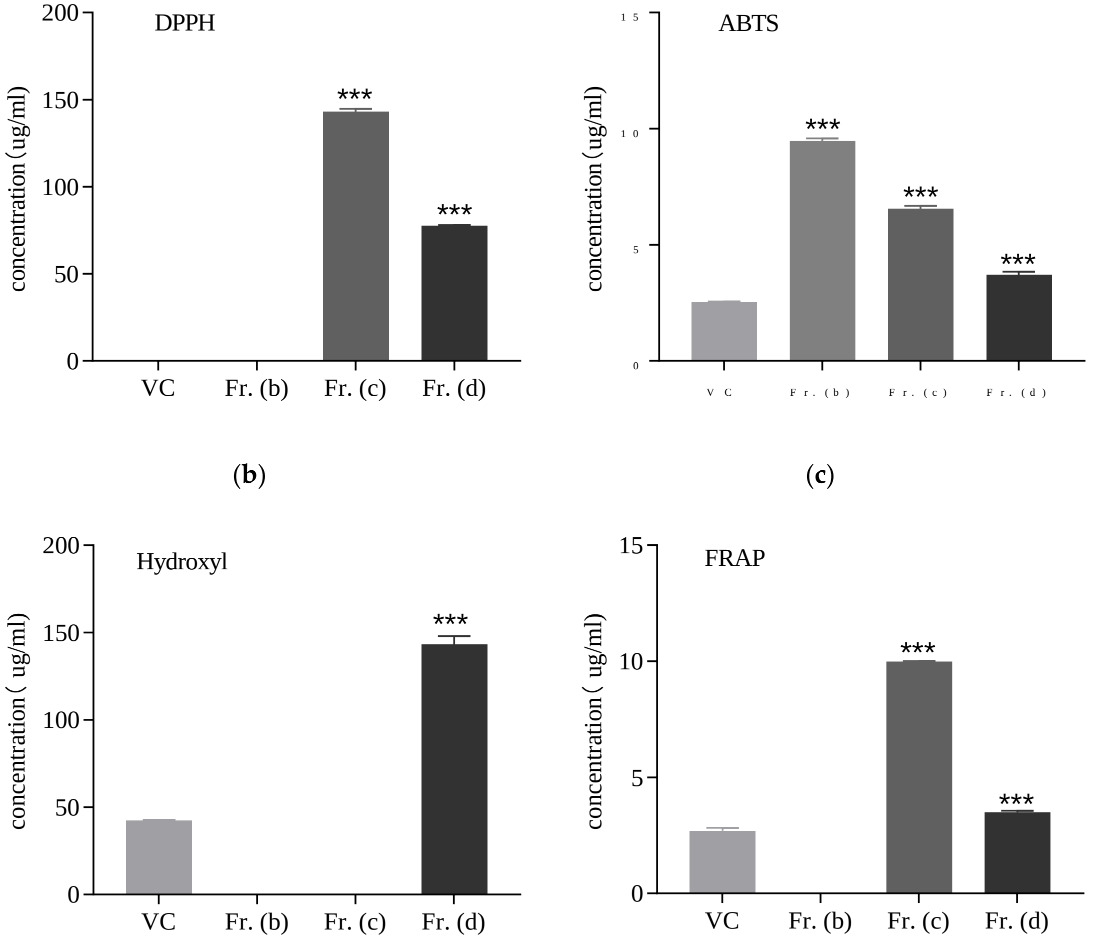

Supplement: Supplementary file 1 [file foods-13-03116-s001.zip › foods-3211231-supplementary.pdf]
